# Do cultural and linguistic competence matter in Latinos’ completion of mandated substance abuse treatment?

**DOI:** 10.1186/1747-597X-7-34

**Published:** 2012-08-16

**Authors:** Erick G Guerrero, Michael Campos, Darren Urada, Joy C Yang

**Affiliations:** 1 School of Social Work, University of Southern California, 655 West 34th Street, Los Angeles, CA, 90089-041, USA; 2 Gambling Studies Program, University of California, Los Angeles, 760 Westwood Plaza, Suite 38-260, Los Angeles, CA, 90024, USA; 3 Integrated Substance Abuse Programs, Semel Institute for Neuroscience and Human Behavior, University of California, Los Angeles, 11075 Santa Monica Blvd., Suite 200, Los Angeles, CA, 90025, USA; 4 Center for Chinese Studies, University of California, Los Angeles, 11381 Bunche Hall, Los Angeles, CA, 90095-1487, USA

**Keywords:** Cultural and linguistic competence, Treatment completion, Mandated substance abuse treatment, Latinos

## Abstract

**Background:**

Increasing evidence suggests that culturally and linguistically responsive programs may improve substance abuse treatment outcomes among Latinos. However, little is known about whether individual practices or culturally and linguistically responsive contexts support efforts by first-time Latino clients to successfully complete mandated treatment.

**Methods:**

We analyzed client and program data from publicly funded treatment programs contracted through the criminal justice system in California. A sample of 5,150 first-time Latino clients nested within 48 treatment programs was analyzed using multilevel logistic regressions.

**Results:**

Outpatient treatment, homelessness, and a high frequency of drug use at intake were associated with decreased odds of treatment completion among Latinos. Programs that routinely offered a culturally and linguistically responsive practice—namely, Spanish-language translation—were associated with increased odds of completion of mandated treatment.

**Conclusions:**

These preliminary findings suggest that concrete practices such as offering Spanish translation improve treatment adherence within a population that is at high risk of treatment dropout.

## Background

The Substance Abuse and Crime Prevention Act (SACPA), passed in California in 2000, mandated drug treatment in lieu of incarceration for nonviolent first- and second-time drug offenders. The percentage of Latino clients in SACPA has consistently increased over time [1], and Latinos represent the second-largest ethnic group among program clients [2]. Notably, many Latino clients are entering drug treatment for the first time under SACPA [3]. Due to significant legal implications (e.g., incarceration) for failure to satisfy treatment requirements, it is of critical importance to identify effective substance abuse treatment (SAT) interventions for Latinos in SACPA and other mandated SAT programs.

Reviews of mandated or coerced SAT support the general effectiveness of such treatment and suggest that legal pressure increases client retention and treatment completion [4,5]. Previous research has also indicated that criminal justice referrals to drug treatment can enable treatment program completion, particularly among minority groups [6-8]. However, client intake characteristics such as having a lengthy criminal history, lower treatment motivation, more severe employment, and psychiatric problems as well as drug use at intake have been found to be associated with dropout from court-mandated SAT [9]. Factors that may contribute to higher completion rates for mandated SAT in some cases include the administrative and individual monitoring offered by the criminal justice system through drug and probation courts [9,10]. Court-supervised SAT can prove effective [7,8] but is contingent upon client engagement and motivation to remain in treatment [6]. Adapting existing SACPA treatment practices with culturally responsive services may increase treatment effectiveness and facilitate client motivation, potentially resulting in increased treatment completion.

Treatment completion is likely to be increasingly employed as a measure of program performance given new health care reform legislation [11,12]. In large administrative data sets, treatment completion reflects both achievement of treatment goals at the client level and program performance at the system level. Most studies on health disparities in the United States have focused on client-level factors: differences in client demographics, primary substance used, and addiction severity [11,13,14]. Findings from the national Treatment Episode Data Set [8] have identified seven client characteristics related to successful treatment completion in outpatient SAT: (1) non-Latino White race, (2) female gender, (3) older than 40 years, (4) more than 12 years of education, (5) employment, (6) use of alcohol as primary substance, and (7) less than daily substance use at admission. Referral source was included as a service factor and found to be related to treatment completion, highlighting the need to examine program factors that may impact client response to treatment.

The need to develop an evidentiary base for what constitutes quality of care for racial and ethnic minority clients is recognized [15-18], but little attention has been paid to treatment practices that affect treatment completion in specific racial and ethnic groups entering treatment for the first time [11,19,20]. It is clear that shorter treatment duration and unmet service needs result in lower completion rates for African Americans and Latinos [13,20-24], whereas lack of economic resources is more often associated with poor treatment completion among Whites [8,13].

Data on drug treatment outcomes for Latinos show significantly shorter treatment duration and lower completion rates [25,26] compared to other racial/ethnic groups. This may be partly due to Latinos’ accessing programs with poor quality of care [15,16,22]. High-quality care that engages the often bilingual and bicultural Latino population is generally present in programs with adequate service resources (social service availability and intensity), services in Spanish, and services provided by staff skilled in cross-cultural counseling [15,17,18,27,28]. Cultural and linguistic competence is broadly defined as “a set of congruent behaviors, knowledge, attitudes, and policies that come together in a system, organization, or among professionals that enables effective work in cross-cultural situations” (Cross et al., 1989, p. 13) [18]. In treatment organizations, culturally and linguistic competence is generally operationalized as a set of service-related practices through which organizations recognize and respond to the needs of culturally diverse populations [19]. Such practices generally incorporate clients' native language, cultural nuances and family and community dynamics [19]. Emerging evidence suggests that a program’s ability to introduce the linguistic, cultural, familial, and community norms of Latinos in treatment is associated with greater access, duration, and treatment completion rates [19,27,29,30].

This manuscript builds on previous findings [30] regarding higher completion rates among Latinos referred to publicly funded drug treatment by identifying culturally responsive program practices linked to treatment completion. The goal of this study is to test the evidentiary basis for the use of culturally and linguistically responsive practices that aim to facilitate successful completion of mandated SAT among first-time Latino clients. Findings from this study may support evidence-based practices for Latinos involved in the criminal justice system, resulting in a reduction in health disparities among this underresearched minority population [31].

Experts in the field indicate quality of care for Latinos can be represented by providers' Spanish-language proficiency and knowledge of cultural nuances associated with service provision. This culturally responsive care may strengthen the therapeutic alliance necessary to increase treatment adherence [15,32]. However, provision of culturally and linguistically responsive services in SAT varies a great deal, particularly in terms of service comprehensiveness and intensity [28]. Although emerging evidence suggests that single practices, such as matching counselors and clients based on language and ethnicity, benefit Latinos and African Americans [33], programs that incorporate several culturally and linguistically responsive practices are believed to have a cumulative impact on treatment outcomes among minorities [19]. Thus, Hypothesis 1 posited that Latinos who receive SAT services from SACPA providers with the highest implementation of culturally competent practices will be most likely to successfully complete treatment. Considering that up to 76% of Latinos in California report speaking Spanish at home [34], we expect that specific linguistically competent practices will support Latinos' efforts to complete treatment. Thus, Hypothesis 2 posited that Latinos who receive SAT services from SACPA providers that offer (1) Spanish-speaking counselors, (2) Spanish-language translation, (3) Spanish-language program materials, or (4) Spanish-language health education will be most likely to successfully complete treatment.

## Methods

### Data source and procedures

Data in this study came from two sources: client-level data from the California Outcomes Measurement System (CalOMS) and program-level data from a 2008 survey. CalOMS is a mandatory data collection and reporting system for all publicly funded alcohol and drug programs in California. CalOMS data are reported to the state’s Department of Alcohol and Drug Programs (ADP). Data domains for CalOMS include drug and alcohol use, criminal justice involvement, employment, education, family/social functioning, and physical/mental health. The program-level data were gathered via a survey designed and distributed by University of California, Los Angeles, research staff as part of the ADP-funded statewide SACPA evaluation. This survey assessed program/treatment characteristics, treatment services, treatment populations, and performance measures. Survey respondents received a letter of thanks and a $75 money order as compensation for their participation. The study was approved by both university and state institutional review boards.

### Participants

Surveys were distributed in June 2008 to a random sample of 105 Proposition 36 providers serving more than five clients in the year prior to the survey as indicated by CalOMS records. A total of 67 programs responded, resulting in a response rate of 63.8%. Four programs indicated that they were no longer Proposition 36 providers. Thus, the final survey sample included 63 providers from 25 counties. Although this response rate is somewhat low, it is still significant given the dearth of knowledge in this area of program evaluation [35].

We placed a number of restrictions on the CalOMS sample to increase the rigor of our statistical analysis. First, we restricted the sample to eligible clients admitted and discharged in fiscal year 2007–2008 (July 1 to June 30). Second, because we were specifically interested in the association between the cultural and linguistic competence of providers and Latinos' rate of treatment completion, we restricted the sample to Latinos. Third, although some clients were treated more than once during this period, only the first treatment episode was used in the current study to capture client experience at baseline. This sampling strategy has been used in other large studies [11,30]. The final CalOMS analytic sample consisted of 5,150 clients from 48 alcohol/drug treatment facilities across California. To the best of our knowledge, this is one of only a few studies to examine cultural competence using program and client data from one of the largest SAT systems in the United States.

### Measures

#### **Treatment completion**

Using CalOMS data, discharge status was coded into one of four categories: (1) successfully completed treatment/recovery plan, (2) left before completing treatment/recovery plan but made satisfactory progress, (3) left before completing treatment/recovery plan with unsatisfactory progress, or (4) did not complete treatment due to death, incarceration, or other reasons. These four categories were dichotomized (1 = successfully completed treatment on-site, 0 = left with satisfactory progress, left with unsatisfactory progress, or did not complete treatment). This measure is congruent with the most recent regional [14] and national [8] studies. See variable descriptions and response format in Table 1.

**Table 1:** Descriptive statistics and response format

**Variable**	**M (SD) or %**	**Response format**
**Program Variables (n = 48)**		
Culturally competent practices (composite)	10.81(4.60)	Composite of practices listed below
Spanish-speaking counselors	1.86(1.13)	0 = not at all, 1 = limited,
		2 = moderate, 3 = significant
Language translators	0.80(1.06)	0 = not at all, 1 = limited,
		2 = moderate, 3 = significant
Spanish-language material	1.89(1.01)	0 = not at all, 1 = limited,
		2 = moderate, 3 = significant
Spanish-language health education	1.47(1.08)	0 = not at all, 1 = limited,
		2 = moderate, 3 = significant
Client-counselor matching by ethnicity/culture	1.99(0.98)	0 = not at all, 1 = limited,
		2 = moderate, 3 = significant
Incorporation of Latino cultural components	1.36(1.15)	0 = not at all, 1 = limited,
		2 = moderate, 3 = significant
Outpatient treatment	62	1 = successful on-site completion,
		0 = other
**Individual Variables (n = 5,150)**		
Treatment completion	15	1 = successful on-site completion,
		0 = other
Male	69	1 = male,
		0 = female
Homeless	20	1 = unstable housing,
		0 = other
Frequency of drug use	9.78(12.03)	Days of primary drug use prior to treatment
Number of treatment episodes	1.45(2.68)	Range = 1-12

#### **Program-level variables**

Using common program-level measures of cultural and linguistic competence [19], the following practices were measured: (1) having Spanish-speaking counselors, (2) using language translators when/if necessary, (3) offering program materials in Spanish, (4) providing health education in both Spanish and English, (5) matching client and counselors on ethnicity/culture if requested by client, and (6) incorporating Latino cultural components in treatment. Each of the six practices was rated on a 4-point Likert scale (*not at all*, *to a limited extent*, *to a moderate extent*, and *to a significant extent*). Our main independent variables included these six practices, as well as a composite variable measured by summing all six scores.

#### **Client-level variables**

In previous research on health disparities, differences in treatment completion across groups are most often attributed to differences in individual characteristics such as employment, family background, and addiction severity [14]. Nationally representative data has indicated seven client-level factors associated with a higher likelihood of successful treatment completion in outpatient treatment: (1) alcohol as a primary substance; (2) employment; (3) less than daily substance use at admission; (4) female gender; (5) being older than 40; (6) having more than 12 years of education; and (7) non-Latino White background [8]. Our work focused on program-level factors, after controlling for some individual-level factors, in order to determine program components that may facilitate improved treatment outcomes for Latinos in mandated drug treatment. Significant differences in relation to treatment completion among Latinos have been noted with respect to gender, drug use severity (days of primary drug use before admission) and number of treatment episodes [30]. As such, these variables were included in the analysis.

### Statistical analysis

To take advantage of the maximum amount of information in the dataset, multiple imputation was used to fill in missing values, which reached 10% in some measures. The assumption of data missing at random was supported by showing that the probability of having a missing value for the main explanatory variables was not associated with the dependent variable [36]. The Markov Chain Monte Carlo method [37] was used to generate five possible values for each missing value and increase the accuracy in parameter estimation.

To assess the association between multilevel explanatory variables and individual treatment completion rates, we conducted multilevel logistic regressions for the analytical sample via the SAS command PROC GLIMMIX. This procedure relies on random intercept models that account for the hierarchical structure of the data (clients nested within programs), as suggested in other multilevel analyses in addiction health services research [22,33,38]. By specifying random effects at each level of analysis, multilevel modeling takes the hierarchical structure of data into account and provides more conservative inferences [39]. We also modeled the predicted probability of treatment completion for each practice level of implementation. For instance, for program offering translation in Spanish language, we modeled the predicted probability of treatment completion for program reporting the following four levels: *not at all*, *to a limited extent*, *to a moderate extent*, and *to a significant extent*), holding all other variables in the model at their means. Results from this analysis is only presented in the narrative.

## Results

Table 2 presents two final random effects logistic regression models showing that statistically significant variance exists in levels of cultural competence among SAT programs. Model 1 tests Hypothesis 1 with a composite measure representing the culturally and linguistically responsive context of SAT programs. Model 2 tests Hypothesis 2, examining the effect of single culturally and linguistically responsive practices on treatment completion.

**Table 2:** Random effects logistics regression on treatment completion

**Independent variables**	**Treatment completion**	
	**Model 1**	**Model 2**
	**OR (95% CI)**	
Program Variables (n = 48)		
Culturally competent practices (composite)	1.04(0.95-1.15)	–
Spanish-speaking counselors	–	1.02(0.62-1.66)
Language translators	–	**1.47(1.16-1.88)**
Spanish-language material	–	1.59(0.95-2.68)
Spanish-language health education	–	0.95(0.55-1.63)
Client-counselor matching by ethnicity/culture	–	0.88(0.63-1.22)
Incorporation of Latino cultural components	–	0.83(0.57-1.21)
Outpatient treatment^a^	**0.57(0.39-.082)**	**0.54(0.37-0.79)**
Individual Variables (n = 5,150)		
Male^b^	120(0.99-1.46)	1.22(0.99-1.50)
Homeless^c^	**0.66(0.48-0.91)**	**0.66(0.48-0.91)**
Frequency of drug use	**0.98(0.97-0.99)**	**0.98(0.97-0.99)**
Number of treatment episodes	0.93(0.88-1.01)	0.93(0.88-1.01)
sigma_u	**1.69(1.27-2.28)**	**1.47(1.10-1.97)**
rho	**0.47(0.33-0.61)**	**0.39(0.26-0.54)**
Wald chi-square*	38.22, df = 6	48.43, df = 11

Note. Values in bold are significant at a 95% confidence interval that does not bound 1.

*p > chi-square = .0001, df = degrees of freedom.

^a^Reference is inpatient, hospital, and day treatment.

^b^Reference is female.

^c^Reference is other housing.

Hypothesis 1 was not supported. Model 1 in Table 2 shows that SACPA programs with the highest composite score of culturally and linguistically competent practices were not associated with increased odds of treatment completion among Latinos (*OR* = 1.04; CI = 0.95-1.15).

Partial support was found for Hypothesis 2, which posited that Latinos who receive SAT services from SACPA providers that rely more often on practices such as (1) Spanish-speaking counselors, (2) language translators, (3) program materials in Spanish, or (4) health education in Spanish will be most likely to complete treatment successfully(see Model 2 in Table 2). Only clients attending programs that frequently relied on Spanish-language translators reported higher odds of completing treatment than those in programs that did not offer this practice (*OR* = 1.47; CI = 1.16-1.88). Latinos also reported a higher treatment completion rate when attending programs that offered material in Spanish. But this association was only statistically significant at *p* < .10.

Several individual and program factors were associated with lower odds of treatment completion. Latinos reporting homelessness were associated with the lowest odds of completing treatment (*OR* = 0.66; CI = 0.48-0.91). Similarly, higher frequency of drug use at intake was associated with lower odds of completing treatment (*OR* = 0.98; CI = 0.97-0.99). At the program level, Latinos attending outpatient treatment (compared to other modalities such as inpatient or hospitalization) were less likely to complete treatment as well (*OR* = 0.52; CI = 0.35-0.76). Finally, it is noteworthy to indicate that both models reported significant variability in treatment completion across programs.

## Discussion

The results of this preliminary study show that after accounting for individual and program characteristics, specific linguistically responsive practices play a significant role in successful treatment completion among first-time Latino clients. Although completing treatment was challenging for all clients (as evidenced by an overall completion rate of 15%), clients attending programs that used language translators more often reported a higher percentage of Latino clients completing treatment. Specifically, the predicted probability for Latinos completing treatment in programs offering Spanish-language translators *to a significant extent* was .20, while the predicted probability of completion for programs *not at all* offering this practice was .11. Similarly, for other linguistically responsive practices, although only statistically significant at *p* < .10, the predicted probability of completion was also higher for programs using these practice *to a significant extent* versus *not at all*. This is the case for programs offering material in Spanish-language (*to a significant extent* = .15, *not at all* = .11) and for programs offering health education in Spanish (*to a significant extent* = .16, *not at all* = .10). It is clear that a higher level of implementation of these linguistically responsive practices is associated with a higher predicted probability of treatment completion among Latinos entering treatment for the first time.

The main contribution of this preliminary study was identifying evidentiary support for the use of Spanish-language translators in treatment completion among first-time Latino clients at high risk of dropout. But this finding needs to be interpreted in light of its limitations. We would like to recognize limitations associated with using cross-sectional survey and administrative data. Our analysis was limited to associations among program- and client-level variables, and the data did not include all program and client variables of importance for treatment completion (e.g., service intensity, mental health status). Despite this limitation and unlike other studies, there was sufficient power to identify statistical differences, and independent variables accounted for 42% of the variance explained in the outcome. In addition, although all of the culturally and linguistically responsive practices were correlated with treatment completion at an average of *r* = .10, practices were not correlated with each other beyond *r* = .50. These practices, generally considered indicators of quality of care for this population, together were not found to improve Latinos' response to treatment. To improve treatment adherence among this population entering treatment for the first time, it is also necessary to consider other program and client factors associated with service intensity, quality, and matched service needs [9,11,22,30].

## Conclusions

Overall, this study highlighted the importance of language-based practices that support the treatment engagement of Latinos regardless of their within-group heterogeneity in terms of country of origin, acculturation, generation in the United States, and other individual factors. Although administrative data can pose methodological challenges, these data allowed us to capture the experience of a hard-to-reach population and identify its response to tailored services in the largest substance abuse treatment system in the country. As health care reform begins requiring providers to increase their level of cultural and linguistic competence to respond to the increase of newly eligible Latino clients in California and other states, it is necessary to build upon these studies to establish an evidentiary base for the provision of linguistically competent care in court-mandated treatment.

## Competing interests

Authors declare that they have no competing interest of any kind.

## Authors’ contributions

EG designed the study and conducted the statistical analysis. MC and JY managed the literature searches and summaries of previous related work, contributed to the interpretation of the analysis, and along with DU, helped draft the initial versions of the manuscript. All authors revised several drafts and contributed to and have approved the final manuscript.
